# Microstructure and Corrosion Behavior of ZnAl12Mg3Si0.3 Double-Batch Hot-Dip Coatings

**DOI:** 10.3390/ma16062162

**Published:** 2023-03-08

**Authors:** Henryk Kania, Anżelina Marek, Michał Zoran, Marcin Spławski, Przemysław Kupczyk, Mateusz Wiewióra, Aleksandra Kupczyk

**Affiliations:** 1 Department of Metallurgy and Recycling, Faculty of Materials Engineering, Silesian University of Technology, Krasińskiego 8, 40-019 Katowice, Poland; 2 Faculty of Transport and Aviation Engineering, Silesian University of Technology, Krasińskiego 8, 40-019 Katowice, Poland; 3 Faculty of Civil Engineering, Silesian University of Technology, Akademicka 5, 44-100 Gliwice, Poland; 4 Faculty of Architecture, Silesian University of Technology, Akademicka 7, 44-100 Gliwice, Poland

**Keywords:** hot-dip galvanizing, double hot-dip method, ZnAlMgSi coatings, corrosion resistance

## Abstract

This article presents the microstructure (SEM) and corrosion behavior of ZnAl12Mg3Si0.3 (ZAMS) coatings obtained by the double hot-dip method on Sebisty steel with increased strength. On the basis of chemical composition studies in micro-areas (EDS) and phase composition studies (XRD), structural components of the coating and corrosion products formed on the coating surface after exposure to the neutral salt spray (NSS) test (EN ISO 9227) were identified. The presence of the Fe(Al,Si,Zn)_3_ intermetallic phase was found in the Fe-Al intermetallic layer, while in the outer layer, dendrites rich in Al and Zn were identified. In these dendrites, the eutectics of Zn/MgZn_2_ and precipitates of the MgZn_2_ phase and Si were located. The NSS test showed better corrosion resistance of ZAMS coatings compared to conventional zinc hot-dip coatings. The increase in corrosion resistance is due to the formation of favorable corrosion products: simonkolleite—Zn_5_(OH)_8_Cl_2_·H_2_O and hydrozincite—Zn_5_(OH)_6_(CO_3_)_2_, and the presence of the MgZn_2_ phase in the coating, which is more anodic than other structural components.

## 1. Introduction

Hot-dip-galvanized (HDG) coatings are currently one of the most effective and economical corrosion protection methods for steel. Demand for zinc coatings is constantly growing, covering an ever-wider range of products, such as drawing wires [1], iron castings [2], and products from high-strength steel [3]. The HDG process currently consumes more than 50% of the world’s zinc production, with zinc production steadily increasing. At the same time, natural resources of zinc are estimated at a level that allows to meet the growing demand in about 17 years [4]. It is therefore necessary to limit the consumption of zinc. In particular, galvanizing of high-strength steels containing Si in the Sebisty range (0.12–0.22% Si) and high-silicon steels (above 0.22% Si) [5] causes the formation of excessively thick coatings, which leads to an unjustified increase in zinc consumption.

ZnAlMg coatings are an alternative to zinc hot-dip coatings. These coatings show 2–4 times better corrosion resistance compared to conventional zinc coatings [6]. The increase in corrosion resistance allows to reduce the thickness of the coating, which leads to lower consumption of zinc. An important economic aspect also results from the replacement of zinc in the bath with less expensive metals—aluminum and magnesium, with a lower specific gravity. The introduction of Al and Mg into the coating therefore reduces the weight of the material to form a coating of the same thickness. Coatings such as Super Dyma (Zn-11Al-3Mg-0.2Si) [7], ZAM (Zn-6Al-3Mg) [8], MagiZincTM (Zn, 1–2% Mg, 1–2% Al) [9], and Magnelis (Zn-3.5%Al-3%Mg) [10] have been manufactured for several decades using the batch HDG method on steel sheets.

However, ZnAlMg coatings have many limitations when using the batch hot-dip galvanizing method. The main limitation of their application is the lack of an appropriate flux [11,12,13], excessive dissolution of iron in the bath [14], excessive growth of the diffusion layer of the coating, and the formation of a periodic layered structure [15].

Increasing the Al content in the Zn bath increases the corrosion resistance of the coating [16]. However, it also increases the melting point of the ZnAl alloy [17] and the need to carry out the process at a higher temperature. Studies have shown that the addition of Mg can reduce the melting point of the ZnAl alloy [18]. The ZnAl12Mg3 alloy showed an initial solidification temperature of 414.1 °C, while the two-component ZnAl15 alloy—445.8 °C [19]. Replacing 3 wt.% Al with the addition of magnesium will allow the hot-dip process to be carried out at the temperature of the conventional HDG process. An effective protection against the formation of coatings of excessive thickness and the formation of a periodic layered structure is the addition of Si. Mendala [20] showed that the addition of Si to the ZnAl bath stabilizes the structure of the coating obtained by the batch hot-dip method even at high temperatures and a long immersion in the bath.

In the batch hot-dip process, the long immersion time causes excessive iron dissolution in the ZnAl bath. The excess of iron causes precipitation of Fe-Al intermetallic phase particles, which float on the surface of the bath [14]. The ZnAl bath quickly loses its technological properties, and a further hot-dip process is impossible. An effective method of producing ZnAl coatings at low temperatures using conventional fluxes is the double hot-dip method [21]. In this method, a ZnAl coating is produced on a pre-formed zinc coating. The formation of the ZnAl coating occurs as a result of the reconstruction of the Fe-Zn intermetallic phases, which contain much less iron than the steel substrate [14]. This allows to limit the amount of iron passing to the ZnAl bath.

This paper presents the results of tests on the microstructure and corrosion behavior of the coating obtained by the double hot-dip method in a ZnAl12Mg3Si0.3 (ZAMS) bath on Sebisty steel with increased strength. It seems that the combination of the synergistic interaction of Mg and Si in the ZnAl12 bath will provide a favorable structure and thickness of the coating as well as high corrosion resistance, while maintaining the conventional process temperature. The production of new ZAMS coatings by the batch hot-dip method with increased corrosion resistance will allow to reduce the thickness of the coating and reduce material costs.

## 2. Materials and Methods

### 2.1. Materials

ZAMS coatings were prepared on samples of high-strength steel HSLA for cold forming (SSAB, Hämeenlinna, Finland). The chemical composition and properties of the steel, according to the manufacturer’s certificate, are presented in Table 1. Test specimens with dimensions of 50 × 100 × 2 mm were cut from a cold-rolled wide steel strip.

### 2.2. Hot-Dip Procedure

Test coatings were produced by the double-batch hot-dip method. Before immersion in the bath, the samples were subjected to acid degreasing in Hydronet-Base solution (SOPRIN S.r.l., Maserada Sul Piave, Italy) for 5 min, etching in 12% HCl solution (Chempur, Piekary Śląskie, Poland) for 10 min, rinsing in water and fluxing in a solution of TakiFlux60 (Dipl. Ing. Herwig GmbH, Hagen, Germany) for 2 min, and drying at 120 °C for 15 min. The coatings were produced on a laboratory stand for hot-dip galvanizing with two resistance furnaces equipped with SiC crucibles with a capacity of 3.2 dm^3^ (Remix S.A., Świebodzin, Poland). First, the samples were immersed in a conventional Zn bath for 60 s. Immediately after removal from the Zn bath (HDG), the samples were immersed in a ZnAl12Mg3Si0.3 bath (ZAMS) for 60 s. The temperature of the HDG and ZAMS baths was maintained at 450 °C. The chemical composition of the bath was determined using the ARL 3460 emission spectrometer (Thermo ARL, Waltham, MA, USA) and is presented in Table 2. After removal from the ZAMS bath, the samples were cooled in the air. Bath markings have been added and clarified.

### 2.3. Characterization Methods

Microstructure and chemical composition studies were performed using Hitachi S-3400 N scanning electron microscopy (SEM) equipped with an energy dispersion spectroscope (EDS) (Hitachi, Tokyo, Japan) and the use of Noran Instruments—System Six (Thermo Fisher Scientific, Waltham, MA, USA).

X-ray phase analysis was performed on a Philips X’Pert 3 X-ray diffractometer (Malvern Panalytical, Malvern, UK) using a lamp with a copper anode (λCuKα = 1.54178 Ǻ), supplied with a current of 30 mA at a voltage of 40 kV, and a graphite monochromator. The recording was made continuously with a step of 0.026° in the range of 2θ from 10 to 90°. The tests of the phase composition of the coatings were carried out on the surface of the flat cross-section, including the phase composition on the surface of the coating, and from the surface of the diagonal cross-section, covering the phase composition over the entire cross-section of the coating. White corrosion products were mechanically removed from the coating surface, and then the phase composition of the white corrosion products, which are in powder form, was determined, as well as the phase composition of the corrosion products on the exposed surface of the corroded coating.

### 2.4. Corrosion Testing Method

The neutral salt spray (NSS) test was performed in a CORROTHERM Model 610 salt spray chamber with a volume of 400 dm^3^ (Erichsen, Hemer, Germany). The test parameters were in accordance with EN ISO 9227 [22]: temperature 35 ± 1 °C, 5% NaCl aqueous solution, pH 6.8–7.2, and mist condensation rate on a flat surface of 80 cm^2^—1.5 ± 0.5 mL/h. The smoothness and changes in the surface of the samples were checked every 24 h. Gravimetric tests were performed after 24, 48, 96, 240, 480, 720, and 1000 h of exposure in the chamber. No corrosion products were removed from the surface of the samples before mass measurement. The final result was the average of five samples of the same type and three measurements for each sample. The corrosion rate was characterized on the basis of determining the unitary mass change according to the following formula: *Δm* = (*m_t_* − *m_o_*)·*S*^−1^, where *m_o_* and *m_t_* represent the mass (g) of the sample before and after exposure time t in the salt spray chamber, respectively, and S (m^2^) is the exposure area of the specimen.

## 3. Results and Discussion

### 3.1. Cross-Section Microstructure of Coatings

The cross-section microstructure of the ZAMS coating was investigated by SEM, EDS, and XRD.

**Figure 1:**
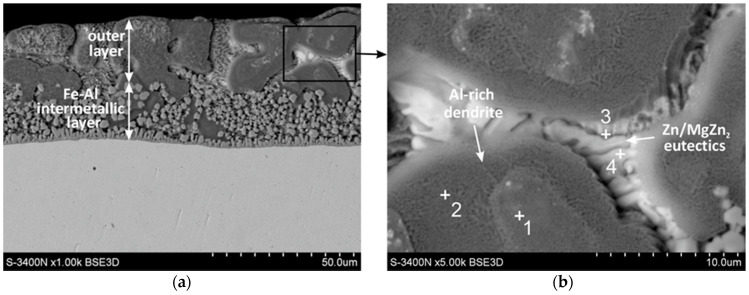
SEM images of the ZAMS coating: (**a**) cross-sectional microstructures and (**b**) Al-rich dendrites and Zn/MgZn_2_ eutectics in the outer layer.

Figure 1 shows the cross-sectional microstructure of the ZAMS coating. From Figure 1a, it can be seen that the coating consisted of a duplex structure (inner layer of Fe-Al intermetallic material and outer layer). The total thickness of the coating was 45.4 ± 2.7 µm. Figure 1b and Table 3 show SEM images and EDS analysis results in the outer layer of the coating. From Figure 1b, it can be seen that the outer layer mainly consisted of Al-rich dendrites and a lamellar eutectic structure in inter-dendritic areas. In the area of Al-rich dendrites, an inner zone (point 1) and an outer zone (point 2) could be distinguished. However, these zones did not show differences in chemical composition. The high content of Al and Zn indicated that Al-rich dendrites were formed by a solid solution of Zn in Al(β). Inter-dendritic spaces were filled with Zn-rich phase (Figure 1b, point 4) and a component rich in Zn and Mg (Figure 1b, point 3). The small volume of structural components did not allow the precise determination of the atomic fraction of the elements by the EDS method. However, the lamellar structure and qualitative contribution of the elements indicated that Zn/MgZn_2_ eutectics was the most probable.

**Figure 2:**
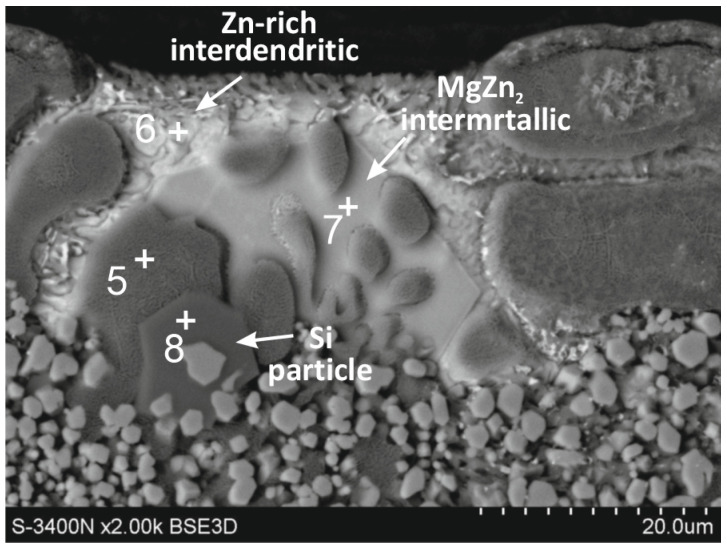
SEM images of the outer layer of the ZAMS coating with MgZn_2_ intermetallic and Si precipitations.

In the structure of the outer layer in the form of Al-rich dendrites and inter-dendrites, the Zn/MgZn2 eutectic was dominant. However, locally, the construction of the outer layer may be more complex. Figure 2 shows SEM images of the outer layer containing locally large inter-dendritic areas with a different morphology. Local EDS analysis of the chemical composition in micro-areas (Table 4) allowed to identify several structural components. The outer layer was formed by Al-rich dendrites (point 5), however, directly in their vicinity, the inter-dendritic areas were filled with Zn-rich phase (point 6). The contents of Al and Zn indicated that these were most likely solutions of Zn in Al (β) and Al in Zn (α), respectively. In the inter-dendritic area, large precipitates containing mainly Mg and Zn were also formed (point 7). The atomic ratio of Zn to Mg was close to 2, which may confirm the presence of the MgZn_2_ intermetallic phase. In the structure of the outer layer, the presence of characteristic, approximately equiaxed Si precipitates could also be distinguished (point 8).

**Figure 3:**
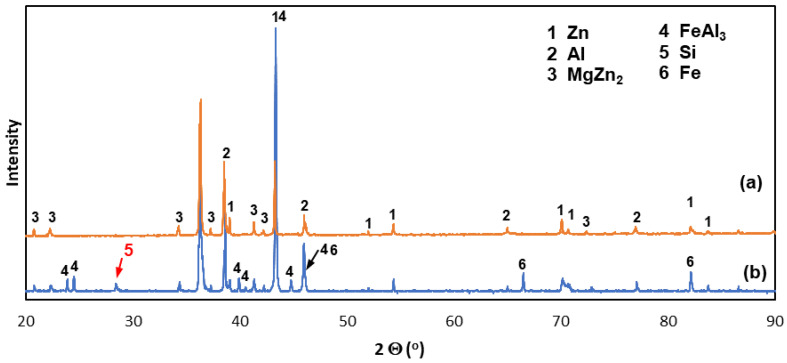
XRD spectra of the ZAMS coating: (**a**) from the flat ground surface of the outer layer of the coating and (**b**) from the bevel cut surface on the cross-section of the coating.

Figure 3 shows the XRD spectra of the ZAMS coating. XRD showed that aluminum, zinc, and MgZn_2_ are present on the flat ground surface of the outer layer of the coating (Figure 3a). The peaks from Al and Zn confirmed the presence of Al-rich dendrites and Zn-rich inter-dendrites in the outer layer of the coating. XRD also confirmed the presence of MgZn_2_ intermetallic coating in the outer layer, which in correlation with SEM and EDS can be located as Zn/MgZn_2_ eutectics in inter-dendritic areas or as separate precipitates. XRD of the coating surface could not confirm the presence of Si precipitates, but XRD from the surface on the cross-section of the coating allowed to identify one independent peak characteristic of Si (marked in red). This, together with the SEM and EDS results, confirmed the presence of Si precipitates in the outer layer as well. The formation of the Si precipitates may indicate the supersaturation of the reaction area with silicon, which is supplied not only from the bath, but also from the dissolving steel substrate. The tested steel contained 0.2% Si (Table 1).

**Figure 4:**
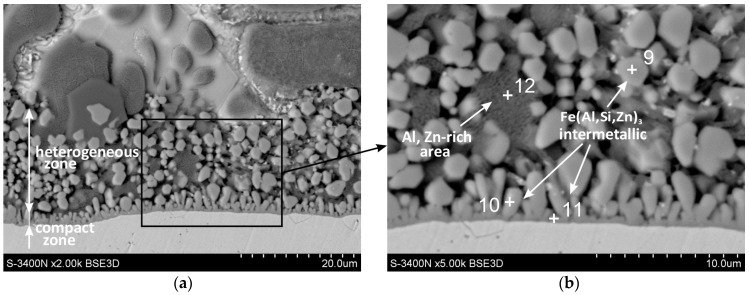
SEM images of the Fe-Al intermetallic layer of the ZAMS coating: (**a**) compact and heterogenous zone of the Fe-Al intermetallic layer and (**b**) analysis points of the EDS of the Fe-Al intermetallic layer.

Figure 4 shows SEM images of the Fe-Al intermetallic layer. This layer consisted of two zones. A thin compact layer was visible at the base, which turned into a zone of heterogeneous structure (Figure 4a). In the heterogeneous zone, regular-shaped precipitates could be distinguished (point 9), between which there was an Al-rich area (point 12) with a composition similar to the Al-rich dendrites observed in the outer layer (Figure 4b). EDS (Table 5) and XRD analysis showed that the precipitates were Fe(Al,Zn,Si)_3_ intermetallics. The XRD pattern from the oblique cut surface (Figure 3b) identified the presence of the FeAl_3_ intermetallic phase on the cross-section of the coating. The Fe-Al intermetallics at point 9 contained 8.5 at.% Si and 10.7 at.% Zn (Table 5). A similar chemical composition could be found in the compact layer at the ground (point 11) and in the precipitates that were in the immediate vicinity of this layer (point 10). Honda et al. [23] found that the coatings obtained in the ZnAlMgSi bath contained Fe-Al intermetallics containing Si and Zn. At the same time, this phase had the same rhombic crystallographic structure as the Fe_2_Al_5_ phase. Ranjan et al. [24] claim that in the Zn-Al21 bath containing the Si addition, the Fe_2_Al_5_ phase is formed, which contains dissolved Si and Zn. However, the tests carried out in the ZAMS bath did not confirm the presence of the Fe_2_Al_5_ phase, but only the presence of the FeAl_3_ phase, which contained both Si and Zn. Many studies [25,26,27,28] indicate, however, that in ZnAlMgSi baths, the initially formed layer of the FeAl_3_ phase may undergo partial or complete transformation into the Fe_2_Al_5_ phase.

One of the important properties of Fe-Al intermetallics is their high concentrations of vacancies, or anti-sites [29]. Point defects control the diffusion-assisted processes that determine the growth of the transition layer of the coatings. The crystal lattice structure of the FeAl_3_ intermetallic has an ideal stoichiometry of 25 at.% Fe and 75 at.% Al. However, the occurrence of lattice defects allows a large range of deviations from the stoichiometric composition. Si and Zn have high solubility in Fe-Al intermetallics in the solid state [30]. Qian et al. [31] showed that at a low Si content in the bath, all Si atoms are dissolved in the Fe_2_Al_5_ phase. Then, the Fe-Al-Si system can be considered as a pseudo-binary system, and the intermetallic phase can be written as Fe_2_(Al,Si)_5_. According to Li et al. [32], the maximum dissolution of Si in the Fe_2_(Al,Si)_5_ phase is 1.40 at.%. This solubility is similar to that of Mirata and Gupta [33]. When the Si content in the bath is greater than 0.2 wt.% and the Fe_2_(Al,Si)_5_ phase reaches the state of saturation with silicon, a three-component τ_4_ (Al_3_FeSi_2_) phase with a much higher Si content is formed in the coating.

Li et al. [32], citing [34], state that this phase contains 46.0–53.5 at.% Al, 16 at.% Fe, and 30.5–38 at.% Si, which is confirmed by the results of EDS research. In the tested ZAMS coatings, it was found that Fe-Al intermetallics contained 8.5 at.% Si (point 9), 8.3 at.% Si (point 10), and 7.7 at.% Si (point 11). These contents exceed the silicon saturation state of the Fe_2_(Al,Si)_5_ phase. The Si content in Fe-Al intermetallics is also much lower than the Si content in AlFeSi ternary phases [34]. Studies by Mirat and Gupta [33] show, however, that the FeAl_3_ phase can dissolve more Si than the Fe_2_Al_5_ phase.

The mechanism of the interaction of Si on Fe-Al intermetallic growth is known mainly from the description of reactions between Fe and the AlSi bath. It is believed that Si occupies a large number of vacancies in the crystallographic lattice of the Fe_2_Al_5_ phase [35], which blocks easy aluminum diffusion paths in this phase [36]. If the structural vacancies of the FeAl_3_ phase after reaching saturation were completely occupied by Si atoms, the atomic ratio (Al + Si)/Fe should increase above 3.0. The presence of large precipitates of Si in the coating in the area of EDS analysis (point 8) may suggest that with the content of approximately 8.5 at.% Si, a state close to saturation was reached, and no more Si could dissolve in the precipitates of the FeAl_3_ phase (point 9). Tests have shown, as shown in Table 3, that the (Al + Si)/Fe atomic ratio is always less than 3.0. At the same time, the total content (Al + Si + Zn) is always constant and close to 75 at.%, which with the Fe content close to 25 at.% (as shown in Table 3) yields the atomic ratio (Al + Si + Zn)/Fe very close to 3.0. This may suggest that Si and Zn atoms replace Al atoms. This is also confirmed by the relatively good agreement of the FeAl_3_ phase peaks in the XRD pattern (Figure 3b). However, it cannot be completely ruled out that the placement of Si atoms also occurs in the structural vacancies of the FeAl_3_ phase. Assuming the replacement of Al atoms by Si and Zn, the phase can be written in accordance with the one proposed by Qian et al. [31], as Fe(Al,Si,Zn)_3_. When Al atoms are replaced by Si and Zn atoms, it will reduce the diffusion rate of Al in the Fe(Al,Si,Zn)_3_ layer. The decrease in diffusion is most likely also caused by the Fe deficit as the Fe-Al intermetallic layer is formed as a result of the remodeling of the pre-formed zinc coating composed of the phases of the Fe-Zn system—δ_1_ and ζ, which contain only 6–11 at.% Fe [37]. The iron deficiency probably also influences the formation of the heterogeneous zone of the intermetallic layer. However, this is advantageous as it inhibits the rapid growth of the coating thickness and the formation of a periodic layered structure.

### 3.2. Corrosion Resistance Determined via NSS Test

**Figure 5:**
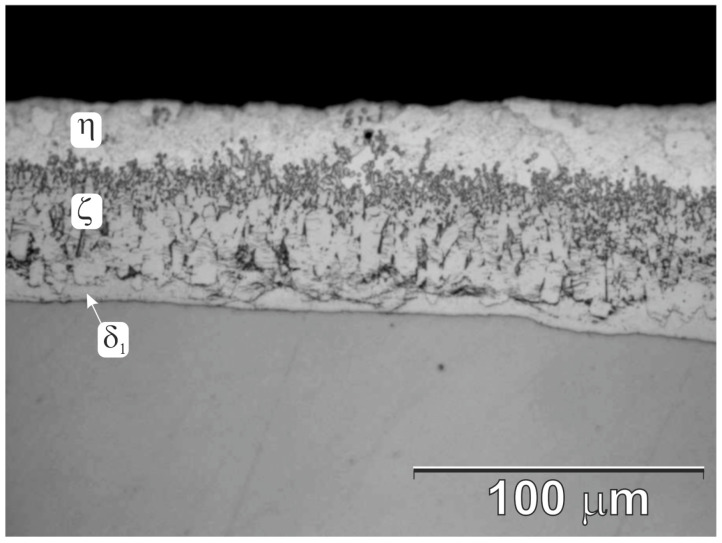
The structure of the zinc hot-dip coating used as a comparative coating in the NSS test.

The corrosion resistance of the coatings obtained in the ZAMS bath was determined on the basis of the mass change rate in the NSS test according to EN ISO 9227. The tested coating was compared in the same corrosion test with conventional zinc hot-dip coatings. The zinc coating was prepared in a Zn bath with the composition shown in Table 2. The coating was prepared on a sample of the same HSLA steel grade at a temperature of 450 °C and an immersion time of 180 s. The average thickness of the zinc coatings was 76.69 ± 5.1 µm. Figure 5 shows the structure of a comparative zinc coating. The zinc coating is made of phases of the Fe-Zn system—δ_1_, ζ, and η. Its structure is characteristic and typical for coatings obtained on Sebisty steel [5].

**Figure 6:**
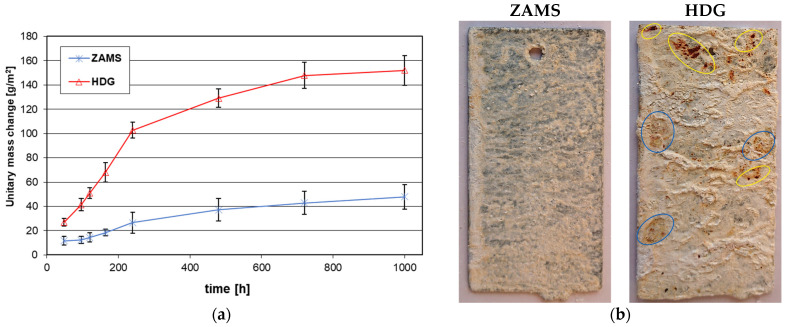
Results of the NSS test: (**a**) development of unit mass change of coatings and (**b**) surface appearance of coatings after the 1000 h NSS test.

Figure 6a shows the average unitary weight changes of samples with ZAMS and HDG coatings during the NSS test. During exposure in NSS, both the Zn coating and the ZAMS coating showed mass gains. The intensity of the mass increases decreased with the lengthening of the corrosion test time. During the NSS test, much greater weight gains of the zinc coating were observed. After 1000 h in the NSS unit test, the weight gain of the coating was 47.82 ± 10.01 g/m^2^ for the coating obtained in the ZAMS bath and 151 ± 12.26 g/m^2^ for the comparative zinc hot-dip coating, respectively. The average unitary weight change of the ZAMS coating after the NSS test was over three times lower than the zinc hot-dip coating.

Figure 6b shows the surface appearance of the ZAMS coatings and the comparative zinc hot-dip coating after 1000 h of exposure in a salt chamber. After completion of the corrosion test, no penetration of the ZAMS coating to the substrate was found. On the other hand, zinc coatings showed clear penetration to the substrate (marked in yellow). In addition, the zinc coating showed a much greater amount of white corrosion products on the surface than the ZAMS coating. White corrosion products are zinc or aluminum corrosion products, as defined in the literature [38]. The presence of rusty discolorations on the zinc coating surface (marked in blue) is also characteristic of the corrosion of Fe-Zn intermetallic phases [39], being a component of these coatings. Such corrosion was not observed on the surface of the ZAMS coating. Corrosion penetrated the zinc coating in this corrosion test, although its average thickness was about 1.7 times greater than the thickness of the ZAMS coating. Thus, the corrosion of the ZAMS coating in an environment containing chlorides proceeds at a much slower rate.

### 3.3. Corrosion Products’ Characterization

**Figure 7:**
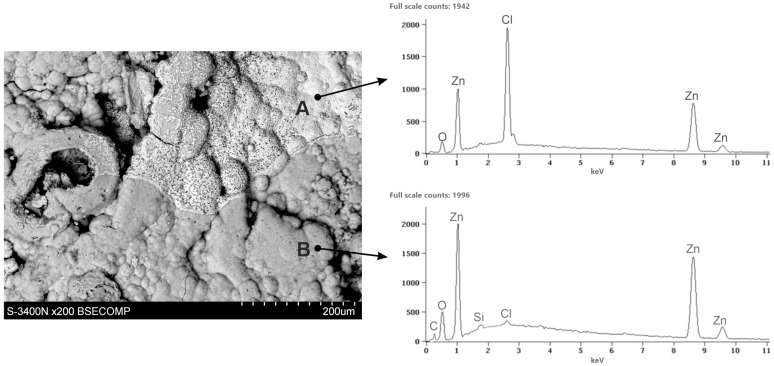
SEM images and corresponding EDS patterns of the corroded ZAMS coating surface after 1000 h exposure in the NSS test.

Figure 7 shows the SEM images of the corroded coating surface (corrosion products) which was exposed to the NSS test for 1000 h. The structure of the corrosion products showed two distinct zones. The EDS spectrum in the bright zone (point A) confirmed the presence of Zn, Cl, and O. In the dark zone (point B), however, a much lower content of Cl, a higher content of Zn, and a small content of Si were found. The EDS spectrum also showed a much stronger oxygen peak in this region, as well as a carbon peak.

**Figure 8:**
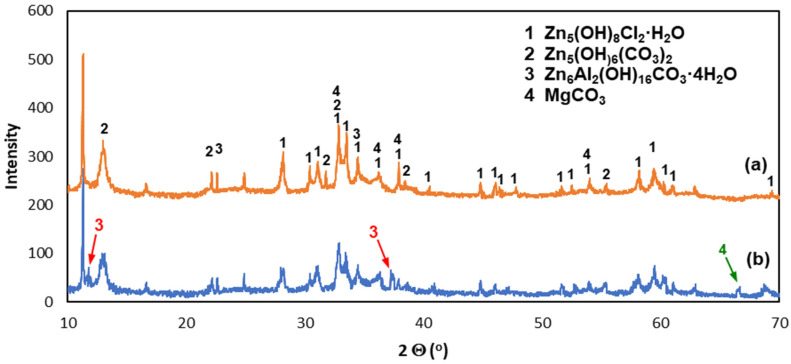
XRD spectra of corrosion products: (**a**) removed from the coating surface and (**b**) the coating surface after removal of white corrosion products.

The XRD pattern of powdered white corrosion products is shown in Figure 8a. According to the presented results of phase composition tests, there were two clearly identified corrosion products in the powdered corrosion products: simonkolleite—Zn_5_(OH)_8_Cl_2_·H_2_O and hydrozincite—Zn_5_(OH)_6_(CO_3_)_2_. Simonkolleit contains chloride, so based on the results of the EDS analysis, it can be concluded that it forms a bright area (point A) of corrosion products. In contrast, hydrozincite does not contain chlorine, but does contain carbon. The EDS analysis therefore indicated that it occurred in the dark region (point B).

**Figure 9:**
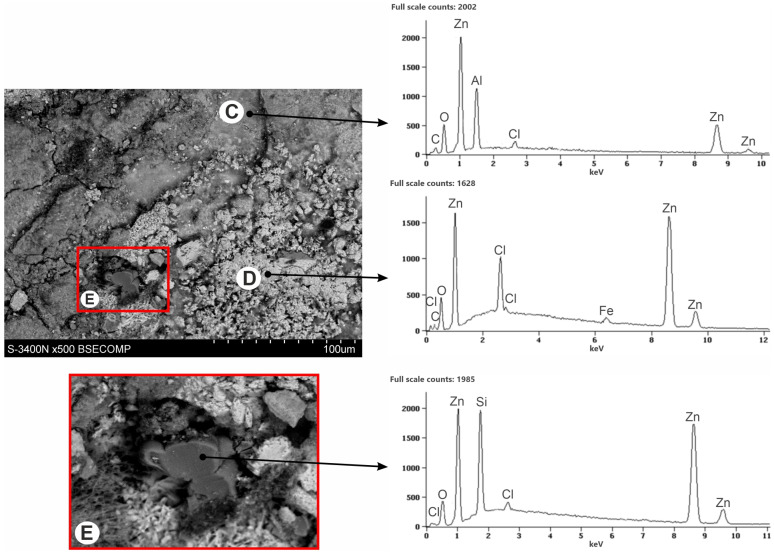
SEM images and corresponding EDS patterns of the corroded ZAMS coating after the removal of white corrosion products.

After mechanical removal of white corrosion products from the surface of the coating, the heterogeneity of its structure can be observed (Figure 9). The EDS X-ray microanalysis performed on the surface of the coating indicated the differentiation of the chemical composition on the surface of the coating. The differences in appearance come from other types of corrosion products being exposed in different micro-areas. The white areas defined in point D are characterized by a high content of Cl and Zn, but also showed an O content. The dark areas, defined in point C, showed a more complex chemical composition. In these areas, the Cl content decreased, the Al content increased, while the Zn content was also high. EDS analysis allowed to identify larger amounts of O and C.

**Figure 10:**
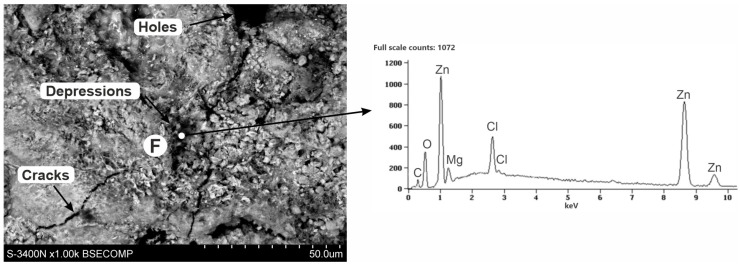
SEM images and corresponding EDS patterns of the corroded ZAMS coating after the removal of white corrosion products with visible cracks, depressions, and holes.

The XRD pattern from the coating surface after mechanical removal of white corrosion products is shown in Figure 8b. There was a clearly identified simonkolleite corrosion product on the surface of the coating—Zn_5_(OH)_8_Cl_2_·H_2_O—which, compared to the EDS microanalysis results, allowed us to say that these are white areas on the coating surface containing Zn, Cl, and O. Simonkolleite is easily rebuilt into hydrozincite—Zn_5_(OH)_6_(CO3)_2_ [40], and hence its presence in this area was also probable. This may be evidenced by the carbon content on the EDS pattern at point D. The presence of zinc aluminum carbonate hydroxide—Zn_6_Al_2_(OH)_16_CO_3_·4H_2_O—can also be identified with a very high probability in the diffraction pattern. The XRD spectra revealed two standalone characteristic spectra of this compound (marked in red) and two characteristic spectra superimposed on the spectra of other identified compounds. According to the results of the EDS microanalysis, these may be darker areas (point C) in the SEM image that contain Zn, Al, C, and O. SEM images also revealed the presence of Si precipitates (area E). XRD spectra were not able to confirm them because their amount in the coating was small. SEM images (Figure 10) of the surface after the removal of white corrosion products also showed the occurrence of cracks, depressions, and holes. The EDS analysis performed in such a cavity (point F) confirmed the presence of Zn, Cl, O, and C, but also Mg. Most likely, these are areas of MgZn_2_ intermetallic occurrence in the form of eutectics (depressions) or precipitation of this phase (holes). XRD spectra may indicate the likelihood of MgCO_3_. However, only one standalone peak of this compound was detected (indicated in green). The presence of other peaks from MgCO_3_ cannot be unambiguously confirmed because they coincide with the strong spectra from Zn_5_(OH)_8_Cl_2_·H_2_O and Zn_5_(OH)_8_(CO_3_)_2_.

### 3.4. Corrosion Behavior of Coating

Increased corrosion resistance of ZAMS coatings is caused by the formation of protective corrosion products. After the NSS test, there were two main corrosion products on the surface of the coating: simonkolleite—Zn_5_(OH)_8_Cl_2_·H_2_O and hydrozincite—Zn_5_(OH)_6_(CO_3_)_2_. Simonkolleite has a very dense and compact structure [41], while hydrozincite is porous, and its adhesion to the coating surface is poor [42]. Prosek believes that the formation of simonkolleite is preferential in the early stages of corrosion [40]. An increase in the concentration of carbonate ions caused by the dissolution of atmospheric CO_2_ leads to the transformation of simonkolleite into hydrozincite, according to the reactions [43]:

(1) $${\mathrm{CO}}_{2}+2{\mathrm{OH}}^{-}\to {\mathrm{CO}}_{3}^{2-}+H_{2}O$$

(2) $${\mathrm{CO}}_{2}+{\mathrm{CO}}_{3}^{2-}+H_{2}O\to 2{\mathrm{HCO}}_{3}^{-}$$

and [41]:

(3) $${\mathrm{Zn}}_{5}{\left(\mathrm{OH}\right)}_{8}\mathrm{Cl}+2{\mathrm{HCO}}_{3}^{-}={\mathrm{Zn}}_{5}{\left(\mathrm{OH}\right)}_{6}{\left({\mathrm{CO}}_{3}\right)}_{2}+2H_{2}O+2{\mathrm{Cl}}^{-}$$

**Figure 11:**
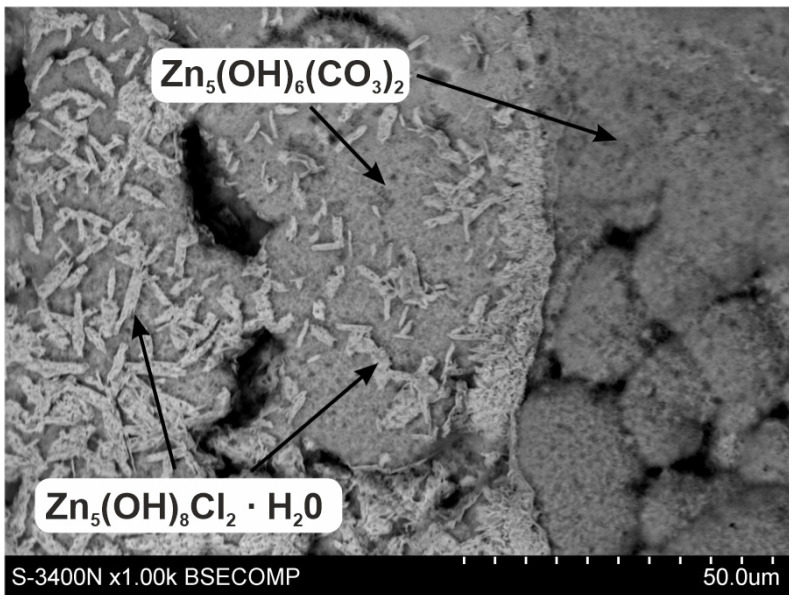
SEM images of the corroded ZAMS coating in the simonkolleite transformation area—Zn_5_(OH)_8_Cl_2_·H_2_O and in hydrozincite—Zn_5_(OH)_6_(CO_3_)_2_.

The SEM image in Figure 11 shows the transformation area on the surface of white corrosion products with visible pores in the hydrozincite layer. After removing the white corrosion products from the ZAMS coating, simonkolleite residues could be clearly seen directly on its surface (Figure 9, point D). This may indicate that the formation of simonkolleite proceeds continuously, and is initiated on the surface of the coating along with the progress of the corrosion process into the coating. The transformation of simonkolleite into hydrozincite is caused by the subsequent interaction of CO_2_ from the atmosphere and progressive changes in the corrosion products themselves.

Studies have shown that under the layers of simonkolleite and hydrozincite, a layer of zinc aluminum carbonate hydroxide—Zn_6_Al_2_(OH)_16_CO_3_·4H_2_O—is formed on the surface of the coating. It is most likely formed on the surface of Al-rich dendrites. Yang et al. [44] report that the Zn_6_Al_2_(OH)_16_CO_3_·4H_2_O layer, which is stable and compact, is the main reason for the high corrosion resistance of galvalume coatings. As a result, the Zn_6_Al_2_(OH)_16_CO_3_·4H_2_O layer can be a passive layer protecting Al-rich dendrites. The corrosion potential of Al in the passive state in most aqueous solutions is higher than in the active state [45]. Therefore, the corrosion potential of Al-rich dendrites is higher than that of Zn-rich inter-dendrites and MgZn_2_ intermetallics (Table 6). This probably provides sacrificial protection of Al-rich dendrites by inter-dendrite areas.

The corrosion process took place mainly in the inter-dendritic area. The formation of holes and depressions was found in it, in which the possibility of the formation of MgCO_3_ was observed, although its presence could be clearly confirmed. Han and Ogle [47] showed that the MgZn_2_ phase dissolved Zn and Mg much more slowly than pure metals. In addition, Mg preferentially dissolved in relation to Zn, which resulted in the formation of protective layers of ZnO and Zn(OH)_2_ on the surface of the MgZn_2_ phase, which slowed down further dissolution of Mg. It is probable that dissolved Mg reacts with the corrosive environment, but also with O and CO_2_ from the atmosphere, according to the reactions [43]:

(4) $$\mathrm{Mg}+\frac{1}{2}O_{2}\to \mathrm{MgO}$$

(5) $$\mathrm{MgO}+H_{2}O\to \mathrm{Mg}{\left(\mathrm{OH}\right)}_{2}$$

and [48]:

(6) $$\mathrm{Mg}{\left(\mathrm{OH}\right)}_{2}+{\mathrm{CO}}_{2}\to {\mathrm{MgCO}}_{3}+H_{2}O$$

According to Li et al. [49], Mg reactions take place preferentially, which reduces the concentration of carbonate ions in corrosion products. This improves the stability of simonkolleite and inhibits its transformation into hydrozincite. As a result, the compactness of corrosion products and their barrier protection are improved.

It seems that the areas rich in Mg were the most active in terms of corrosion in the tested coating. This was also confirmed by the lowest value of the corrosion potential of the MgZn_2_ phase (Table 6), which occurred in Zn/MgZn_2_ eutectics and in the form of precipitates in Zn-rich inter-dendrites. The presence of the MgZn_2_ phase is an additional sacrificial protection mainly for Zn-rich inter-dendrites, but also for Al-rich dendrites.

Si precipitates showed the most cathodic character in the coating. Figure 9 shows matrix dissolution around the Si precipitate. Particularly, large Si precipitates may promote pitting corrosion around them. However, in the ZAMS coating, the amount of Si precipitates was small. In addition, Si tended to form SiO_2_ on the surface, which reduced the effectiveness of the galvanic connection with the matrix [50]. The low cathodic reaction rate on Si due to SiO_2_ limits the impact of Si precipitations on the reduction of corrosion resistance of the coating.

## 4. Conclusions

The microstructure and corrosion behavior of the ZAMS coating obtained by the double hot-dip method on Sebisty steel was tested. The following conclusions can be drawn from the obtained test results:

- The ZAMS coatings obtained by the double hot-dip method on Sebisty steel with increased strength had a duplex structure. The diffusion layer of the coating was made of the Fe(Al,Si,Zn)_3_ intermetallic phase, and the outer layer was Al-rich dendrites and Zn-rich inter-dendrites, in which Zn/MgZn_2_ eutectic and MgZn_2_ intermetallic precipitates were located. Si precipitates were also locally formed in the inter-dendritic areas.
- The ZAMS coatings showed better corrosion resistance than conventional HDG coatings. In the NSS test, the ZAMS coatings showed lower mass gains of corrosion products and did not penetrate the substrate, despite the lower thickness compared to HDG coatings.
- The ZAMS coatings had high corrosion resistance due to the formation of protective corrosion products. In the NSS test, the coating was covered with a layer of simonkolleite—Zn_5_(OH)_8_Cl_2_·H_2_O, which was transformed into hydrozincite—Zn_5_(OH)_6_(CO_3_)_2_. On the surface of Al-rich dendrites, a Zn_6_Al_2_(OH)_16_CO_3_·4H_2_O layer was formed, which may passivate this phase. The presence of Mg in the inter-dendritic areas probably caused the formation of MgCO_3_, which may be the reason for limiting the transformation of the simonkolleite, which protects against further corrosion, into porous hydrozincite.
- The presence of MgZn_2_ intermetallics in the coating can provide sacrificial protection for both Zn-rich inter-dendrites as well as Al-rich dendrites.

## Figures and Tables

**Table 1:** Chemical composition and strength properties of HSLA steel.

Content (wt.%)							Strength Properties		
C	Si	Mn	S	P	Al	Fe and Others	R_e_ (N/mm^2^)	R_m_ (N/mm^2^)	A (%)
0.06	0.20	0.80	0.003	0.009	0.035	rest	465	528	23

**Table 2:** Chemical composition of research baths.

Bath	Bath Designation	Content (wt.%)					
		Al	Fe	Si	Mg	Bi	Zn and Others
Zn	HDG (Hot-Dip Galvanizing)	0.0059	0.031	0.001	0.002	0.061	rest
ZnAl12Mg3Si0.3	ZAMS (Zinc, Aluminum, Magnesium, Silicon)	11.86	0.024	0.32	3.15	0.0001	rest

**Table 3:** Results of the EDS analysis of the outer layer of coatings and the corresponding phase (analysis points as shown in Figure 1b).

Point No.	Mg (at.%)	Al (at.%)	Fe (at.%)	Zn (at.%)	Phase
1	-	82.5	-	17.5	Al-rich phase
2	-	81.7	-	18.3	Al-rich phase
3	7.8	8.7	0.9	82.5	MgZn_2_ intermetallic
4	0.7	6.0	0.6	92.8	Zn-rich phase

**Table 4:** Results of the EDS analysis of the outer layer of coatings and the corresponding phase (analysis points as shown in Figure 2).

Point No.	Mg (at. %)	Al (at. %)	Si (at. %)	Fe (at. %)	Zn (at. %)	Atom Ratio Zn/Mg	Phase
5	-	82.1	-	-	17.9	-	Al-rich phase
6	-	7.1	-	0.4	92.5	-	Zn-rich phase
7	31.77	-	-	-	68.23	2.14	MgZn_2_ intermetallic
8	-	2.0	98.0	-	-	-	Si particles

**Table 5:** Results of the EDS analysis of the outer layer of coatings and the corresponding phase (analysis points as shown in Figure 4).

Point No.	Al (at.%)	Si (at.%)	Fe (at.%)	Zn (at.%)	(Al + Si + Zn) (at. %)	Atom Ratio (Al + Si)/Fe	Atom Ratio (Al + Si + Zn)/Fe	Phase
9	55.6	8.5	25.2	10.7	74.8	2.54	2.96	Fe (Al, Si, Zn)_3_
10	56.2	8.3	24.6	10.9	75.4	2.62	3.06	Fe (Al, Si, Zn)_3_
11	52.6	7.7	25.5	14.2	74.5	2.36	2.92	Fe (Al, Si, Zn)_3_
12	73.8	1.4	4.9	19.9	-	-	-	Al-rich phase

**Table 6:** Main phases detected in the ZAMS coating and corrosion potential in 0.6 M NaCl [46].

Phase	E_corr_ vs. SCE * (mV)
Al	−849
Zn	−1028
MgZn_2_	−1095
Si	−452

* Saturated Calomel Electrode (SCE).
